# The histological rarity of thyroid cancer

**DOI:** 10.1590/S1808-86942012000400010

**Published:** 2015-10-20

**Authors:** Débora Modelli Vianna, Otávio Alberto Curioni, Luciano José de Lemos França, Diógenes Lopes de Paiva, Bernardo Fontel Pompeu, Rogério Aparecido Dedivitis, Abrão Rapoport

**Affiliations:** Resident physician in Otorhinolaryngology and Head and Neck Surgery - Heliópolis Hospital, São Paulo - SP, Brazil; PhD in Pathology - Medical School of the University of São Paulo; Head of the Otorhinolaryngology and Head and Neck Surgery of the Heliópolis Hospital, São Paulo - SP, Brazil; Resident physician in Otorhinolaryngology and Head and Neck Surgery - Heliópolis Hospital, São Paulo - SP, Brazil; MSc in Oncology - Antonio Prudente Foundation; Assistant Physician - Department of Otorhinolaryngology and Head and Neck Surgery - Heliópolis Hospital, São Paulo - SP, Brazil; M.D. Senior Associate Professor - Fundaçã o Lusíada UNILUS; Senior Associate Professor - Medical School of the University of São Paulo (Technical Director - Heliópolis Hospital, São Paulo - SP, Brazil) Department of Otorhinolaryngology and Head and Neck Surgery - Heliópolis Hospital, São Paulo/SP, Brazil

**Keywords:** carcinoma, medullary, lymphoma, neoplasm metastasis, sarcoma, thyroid neoplasms

## Abstract

Thyroid cancer is the most common endocrine cancer, accounting for about 1% of all cancers. Sarcomas, lymphomas and metastases to the thyroid gland are rare and only with a handful of descriptions in the literature.

**Objective**: To describe rare histological types of thyroid cancer found in a reference center.

**Methods**: Medical chart review from admitted patients diagnosed with thyroid cancer in the period from 1977 to 2010. Demographic, diagnostic, therapeutic and histopathological information were collected.

**Results**: 3,018 records of patients admitted with thyroid disease were reviewed. Among the cases diagnosed with rare tumors there was a predominance of: anaplastic carcinoma: 22 cases (0.7%), followed by 11 cases of medullary carcinoma (0.36%); 2 cases of sarcoma (0.07%), 2 cases of lymphoma (0.07%) and one case of metastatic carcinoid tumor (0.03%). There were more females diagnosed (57%) as well as Caucasians (84%). The most frequent clinical presentation was a palpable thyroid nodule. All patients with lymphoma, sarcoma and anaplastic carcinoma died.

**Conclusion**: Sarcomas, lymphomas and thyroid metastases are uncommon and tend to worse outcomes.

## INTRODUCTION

Thyroid cancer is the most common neoplasia of the read and neck, representing 1% of all malignant tumors in the age range between 30 and 74 years, with a three fold higher prevalence in women, when compared to men, although such difference drops after 48 years of age^1^. In a North-American statistics, it corresponds to 3% of all the neoplasia affecting women, there were estimates of 48,020 cases of thyroid cancer in both genders in 2011. According to information from the National Cancer Institute in Brazil, the incidence of new thyroid cancers for 2012, gross rate/100 thousand women, varied between 3.4 and 17.06 among the different geographic regions^2^.

As to histological type, adenocarcinoma is the most frequent type, reaching 90% of all the cases in different series. Sarcomas, lymphomas and metastases to the thyroid gland are not very much described in the literature.

Sarcomas have a described prevalence of approximately 1%, and 15% to 20% of them happen in the head and neck; 0.014% of the sarcomas are primary of the thyroid. Its cytological diagnosis in preoperative tests is not easy, and its cytology may be mistaken with other thyroid neoplastic lesion, such as anaplastic and medullar carcinomas^3^.

Lymphomas have a thyroid prevalence between 0.6% and 5%; there is a clinical past of Hashimoto thyroiditis in 27% to 100% of the cases. There may be Hodgkin and Non-Hodgkin lymphoma, and the B-cell Non-Hodgkin is the most prevalent^4^.

Metastases to the thyroid are not often described, having a low incidence. There are descriptions of metastases from a primary kidney lesion, melanoma, lung, parathyroid, salivary gland and breast cancer^5^. However, in a study carried out in autopsies from patients which cause of death was malignant neoplasia, they reported that metastases to the thyroid gland is usually a terminal event, and it may happen in up to 120 months after the diagnosis of primary neoplasia and, when diagnosed, the patient already has a bad prognosis^6^, ^7^.

This paper aims at doing a descriptive analysis of the rare histological types of thyroid cancer in a tertiary reference center.

## METHODS

This paper was approved by the Ethics Committee in Research of the Institution where it was done.

We carried out a retrospective study of a series of cases, studying 3,018 charts from patients with thyroid disease submitted to a surgical procedure between 1977 and 2010. We collected demographic information associated with the diagnosis, treatment and histopathological results.

## RESULTS

We studied the surgical causes of thyroid disease, encompassing a total of 3,018 cases. Of these, we found histological benign disease in 72% of the cases, the most prevalent was colloid goiter; 21% of the cases were papilliferous tumors; 4.7% follicular neoplasia; undifferentiated-carcinoma was found in 0.73%; and medullary corresponded to 0.36%.

**Figure 1:**
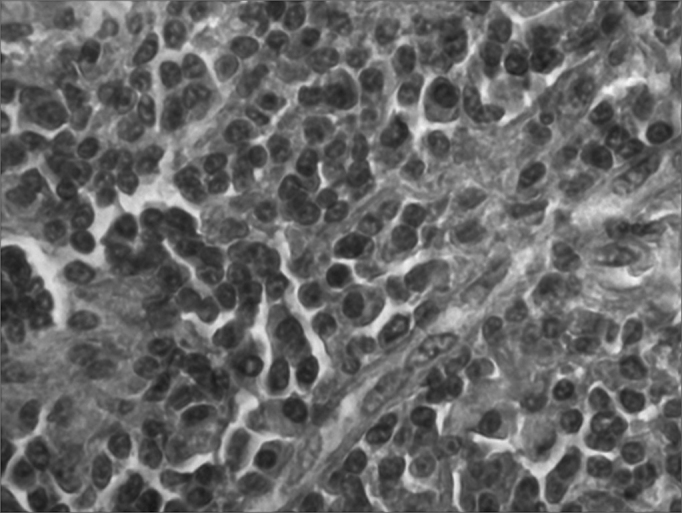
Microphotography of a thyroid lymphoma. HE, 400x.

**Figure 2:**
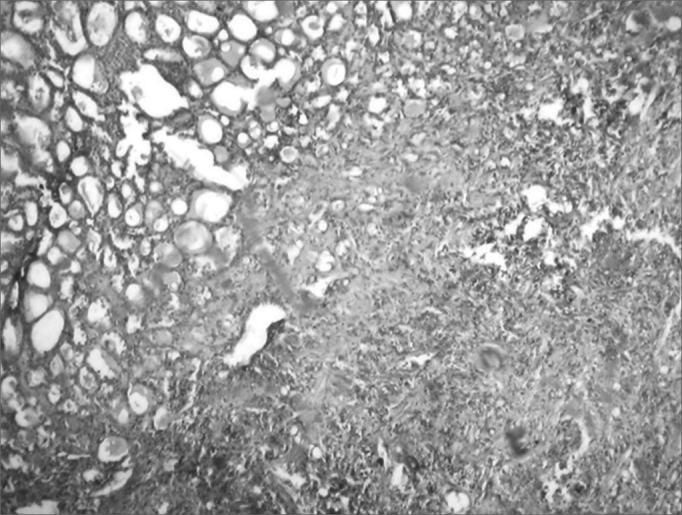
Microphotography of a thyroid fusiform sarcoma. HE, 400x.

Histological types were the lymphomas (Figure 1), sarcomas (Figure 2), undifferentiated carcinomas, medullary carcinoma and metastases to the thyroid gland, which together corresponded to less than 1.30% of the series - Table 1.

**Table 1:** Distribution of cases according to histological type.

Pathology	Number of cases	%
Benign lesions	2194	72.6
Papilliferous carcinoma	645	21.4
Follicular carcinoma	141	4.7
Undifferentiated carcinoma	22	0.73
Medullary carcinoma	11	0.36
Sarcoma	2	0.07
Lymphoma	2	0.07
Metastasis	1	0.07
Total	3018	100

Assessing the series as to neoplastic histology, the result was a predominance of well-differentiated thyroid carcinoma, corresponding to 95% of the cases; followed by undifferentiated carcinomas, 2.7%, and medullary, 1.3% - Table 2.

**Table 2:** Distribution of the cases according malignant histological types.

Malignant neoplasia	Number of cases	%
Papilliferous carcinoma	645	78.3
Follicular carcinoma	141	17.1
Undifferentiated carcinoma	22	2.7
Medullary carcinoma	11	1.3
Sarcoma	2	0.24
Lymphoma	2	0.24
Metastasis	1	0.12
Total	824	100

**Figure 3:**
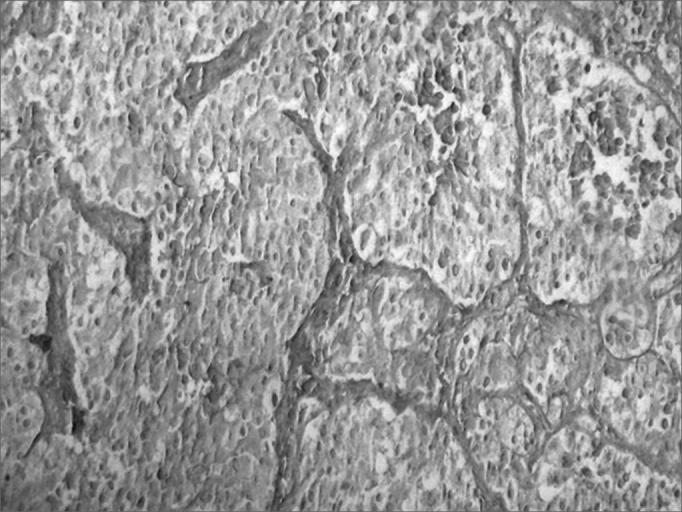
Microphotography of a thyroid carcinoid tumor. HE, 100x.

Concerning the rare types of thyroid cancer, we had 38 cases (4.61%), which most had bad clinical outcomes (Figure 3).

## DISCUSSION

After assessing 3,018 charts of thyroid disease cases operated, we had 95.4% of the patients submitted to surgical treatment for thyroid neoplasia, with histology reports of well-differentiated thyroid carcinoma and only 0.6% of the patients were diagnosed with lymphoma, sarcoma or metastases to the thyroid gland, and such data match those published in the literature^8^.

The undifferentiated carcinoma, contrary to other types of thyroid carcinoma, have a very aggressive behavior, regardless of treatment type, and knowing its clinical and pathological characteristics is very important to establish the best treatment^9^. In our series, it was the most frequent histological type, with a predominance in females (65% of the cases). In general, upon clinical presentation it is possible to make a preoperative clinical diagnosis, because they have symptoms associated to a fast growth neck tumor compressing the upper digestive and air ways. In this series, all the patients died.

Primary thyroid lymphomas have a large spectrum, among which we may distinguish Hodgkin lymphoma and non-Hodgkin lymphoma of large cells, low-grade MALT lymphomas, low-grade B-cell lymphomas with plasmacytic differentiation, Burkitt lymphoma and delta gama lymphoma, and the non-Hodgkin B lymphoma the most frequent lympho-proliferative neoplasia in this gland, as well as B-cell high-grade lymphomas which are more common than the low grade ones^4^.

Primary thyroid lymphomas happen more frequent in women than in men, involving individuals with ages between 50 and 80 years, with an incidence peak in the sixth decade of life; having an annual incidence of two cases per million inhabitants, representing about 2% of the extranodal lymphomas^10^. According to the literature, there is a greater incidence of lymphoma in the cases of autoimmune thyroiditis^11^.

The most common clinical presentation of this type of tumor is similar to that of the thyroid anaplastic carcinoma and is based on a sudden growth in the neck, often times associated to obstructive symptoms^12^. The outcome may be favorable if the diagnosis is early and the treatment is specific.

As to sarcomas, the diagnosis of a primary thyroid lesion requires much prudence, since leiomyosarcoma in the neck are more common than primary thyroid lesions and may invade the gland. By the same token, the primary thyroid lesion may also involve adjacent structures, spilling over the thyroid capsule, which causes diagnostic confusion^3^.

With the help of immunohistochemistry and electronic microscopy, it has been shown that most of the sarcomatous lesions of the thyroid gland are undifferentiated carcinomas with sarcomatous differentiation, with epithelial origin and not mesenchymal, which may show expression of vimentin and cytokeratin, besides the occasional expression of thyroglobulin, but do not show the expression of desmin, muscle-specific actin, chromogranin or calcitonin^13^. Such analysis could not be carried out in a review in our service, since the slides dated of early 80‘s, with compromise of its characteristics.

Concerning metastasis to the thyroid gland, most of the patients are asymptomatic, corroborating its subdiagnosis. Its incidence varies between 1.4 to 4%^7^. Nonetheless, in autopsy studies, the incidence can get to 24%. Although the presentation with thyroid metastatic lesion be considered indicative of a terminal disease, upon detecting thyroid-limited disease, it must be treated surgically. In the case reported in our service, the patient died after the diagnosis.

## CONCLUSION

There are very few described cases of sarcomas, lymphoma and metastases to the thyroid gland. Rare cases of malignant thyroid neoplasia correspond to 0.5% of our series, with a bad clinical outcome.
